# Supplementary material for: Quantifying changes in individual-specific template-based representations of center-of-mass dynamics during walking with ankle exoskeletons using Hybrid-SINDy
Source: Sci Rep. 2024 Jan 10;14:1031. doi: 10.1038/s41598-023-50999-0 (PMC10781730; doi:10.1038/s41598-023-50999-0)
Supplement: Supplementary file 1 — Supplementary Information. [file 41598_2023_50999_MOESM1_ESM.pdf]

Supplemental – S1: Quantifying changes in individual-specific template-based representations of center-of-mass dynamics during walking with ankle exoskeletons using Hybrid-SINDy

Michael C. Rosenberg[1*], Joshua L. Proctor[1,2], Katherine M. Steele[1]

[1]Department of Mechanical Engineering, University of Washington, Seattle, United States of America
[2]Department of Applied Mathematics, University of Washington, Seattle, United States of America

Overview

This supplemental provides details of the spring-loaded inverted pendulum (SLIP) dynamics used in this work, the theory underlying Hybrid Sparse identification of nonlinear dynamics (Hybrid-SINDy), the Akaike Information Criterion (AIC), and an overview of the synthetic SLIP simulations.

1. Derivation of SLIP dynamics
2. The Akaike Information Criterion
3. Comparison to alternative modeling frameworks

SLIP dynamics

SLIP parameters are described in Figure S1.

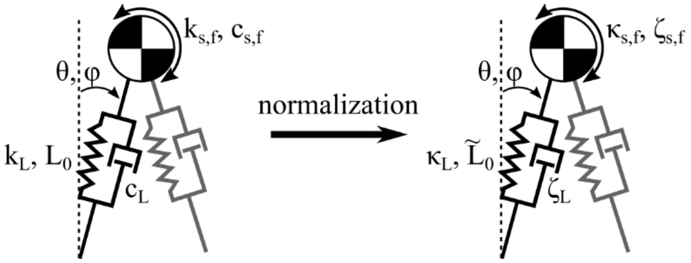

Figure S1: A 2D schematic of the 3D SLIP with model parameters (left) and normalized parameters (right). Variables θ and ϕ denote sagittal and frontal-plane leg angles with respect to vertical, respectively. The center-of-mass (COM) has mass M. Left: Before normalization, model parameters are denoted as: Leg radial stiffness (k_L) and damping (c_L), leg resting length (L_0), rotary stiffness in the sagittal (k_s) and frontal (k_f) planes, and rotary damping in the sagittal (c_s) and frontal (c_f) planes. Non-normalized parameters were estimated for each participant. Right: After normalization, parameters are denoted: Leg radial stiffness (κ_L) and damping (ζ_L), leg resting length ($\tilde{L}_0$), rotary stiffness in the sagittal (κ_s) and frontal (κ_f) planes, and rotary damping in the sagittal (ζ_s) and frontal (ζ_f) planes[1-4]. Normalization was applied to compare participants after model fitting.

We solve the 3D SLIP dynamics with respect to linear accelerations as follows. Let SLIP model mass be denoted by M, and 3D positions of the center-of-mass (COM) relative to the feet be denoted x, y, & z. We solve for linear accelerations of the COM for each foot on the ground as:

$$\begin{aligned} \ddot{x} &= L\ddot{\theta}c\theta - L\dot{\theta}^2 s\theta + 2\dot{L}\dot{\theta}c\theta + \ddot{L}s\theta \\ \ddot{y} &= -L\ddot{\theta}s\theta - L\dot{\theta}^2 c\theta - 2\dot{L}\dot{\theta}s\theta + \ddot{L}c\theta \\ \ddot{z} &= L\ddot{\phi}c\phi - L\dot{\phi}^2 s\phi + 2\dot{L}\dot{\phi}c\phi + \ddot{L}s\phi \end{aligned} \qquad 1$$

where single dots denote first derivatives and double dots denote second derivatives. Other variables are defined as in Figure S1. Note that we assume that anterior-posterior and vertical dynamics are decoupled from frontal-plane dynamics. This simplification is supported by prior studies of COM dynamics during gait[5,6]. We solve radial ($\ddot{L}$) and rotary accelerations ($\ddot{\theta}, \ddot{\phi}$) as a function of spring and damper forcing terms and gravity, g:

$$\begin{aligned} \ddot{L} &= \frac{1}{M}\left[-k_L(L - L_0) - c_L\dot{L}\right] - g\cos(\theta), \\ \ddot{\theta} &= \frac{1}{ML^2}\left[-k_s\theta - c_s\dot{\theta}\right] + \frac{g}{L}\sin(\theta), \\ \ddot{\phi} &= \frac{1}{ML^2}\left[-k_f\phi - c_f\dot{\phi}\right] + \frac{g}{L}\sin(\phi), \end{aligned} \qquad 2$$

where variables correspond to leg radial stiffness (k_L) and damping (c_L), leg resting length (L_0), rotary stiffness in the sagittal (k_s) and frontal (k_f) planes, and rotary damping in the sagittal (c_s) and frontal (c_f) planes. Note that we assume that the resting angle of the rotary stiffness mechanisms is zero[2]. Substituting forcing terms into equation (1) yields:

$$\ddot{x} = \left(\frac{1}{ML^2}\left[-k_s\theta y - c_s\dot{\theta}y\right]\right) - \dot{\theta}^2 x + 2\dot{L}\dot{\theta}\frac{y}{L} + \left(\frac{1}{M}\left[-k_L(L-L_0)\frac{x}{L} - c_L\dot{L}\frac{x}{L}\right]\right)$$

$$\ddot{y} = -\left(\frac{1}{ML^2}\left[-k_s(\theta)x - c_s\dot{\theta}x\right]\right) - \dot{\theta}^2 y - 2\dot{L}\dot{\theta}\frac{x}{L} + \left(\frac{1}{M}\left[-k_L(L-L_0)\frac{y}{L} - c_L\dot{L}\frac{y}{L}\right] - g\right) \qquad 3$$

$$\ddot{z} = \left(\frac{1}{ML^2}\left[-k_f(\phi)y - c_f\dot{\phi}y\right]\right) - \dot{\phi}^2 z + 2\dot{L}\dot{\phi}\frac{y}{L} + \left(\frac{1}{M}\left[-k_L(L-L_0)\frac{z}{L} - c_L\dot{L}\frac{z}{L}\right]\right)$$

Omitting Coriolis terms and adopting the notation $q = [x, y, z]^T$, with vectors and matrices in boldface, we can write the 3D bipedal SLIP dynamics as:

$$M(\ddot{\boldsymbol{q}} + \boldsymbol{g}) = \sum_{j=Right,Left} \left(-\left[k_L(L-L_0) + c\dot{L}\right]\frac{\boldsymbol{q}}{L} - \left[k_s\theta + c_s\dot{\theta}\right]\frac{\boldsymbol{q}}{L_s^2} - \left[k_f\phi + c_f\dot{\phi}\right]\frac{\boldsymbol{q}}{L_f^2}\right)_j, \qquad 4$$

where the summation corresponds to the right and left legs. The left-most brackets contain mechanisms that impart forces radially along the leg: k_L is the leg stiffness, L is the instantaneous leg length, L_0 is the leg resting length, c_L is the leg damping, $\dot{L}$ is the instantaneous leg velocity. The middle bracket contains mechanisms that impart forces transverse to the leg axis in the sagittal plane: k_s and c_s are the sagittal-plane rotary stiffness and damping, respectively. L_s denotes the sagittal-plane leg projection. Analogously in the right-most brackets, k_f and c_f represent the frontal-plane rotary stiffness and damping, respectively, and L_f denotes the frontal-plane leg projection.

Note that we add gravitational acceleration vector, $\boldsymbol{g}$, to both sides of equation (4) to remove it from the right-hand side of the formulation. We can rewrite the dynamics as a linear map from the nonlinear transformations of the SLIP states to linear accelerations: $\ddot{\boldsymbol{q}} = \boldsymbol{\Theta}(\boldsymbol{q}, \dot{\boldsymbol{q}})\boldsymbol{\Xi}$,

$$\ddot{\boldsymbol{q}} + \boldsymbol{g} = \left(\begin{bmatrix} -\frac{k_L}{M} & \frac{k_L L_0}{M} & -\frac{c_L}{M} & \frac{k_s}{M} & \frac{c_s}{M} & \frac{k_f}{M} & \frac{c_f}{M} \end{bmatrix} \begin{bmatrix} x & y & z \\ \frac{x}{L} & \frac{y}{L} & \frac{z}{L} \\ \frac{\dot{L}x}{L} & \frac{\dot{L}y}{L} & \frac{\dot{L}z}{L} \\ \frac{\theta y}{L^2} & \frac{\theta x}{L^2} & 0 \\ \frac{\dot{\theta}y}{L^2} & \frac{\dot{\theta}x}{L^2} & 0 \\ 0 & 0 & \frac{\phi y}{L^2} \\ 0 & 0 & \frac{\dot{\phi}y}{L^2} \end{bmatrix}_{Right,Lleft} \right)^{\mathrm{T}} = \boldsymbol{\Theta}(\boldsymbol{q}, \dot{\boldsymbol{q}})\boldsymbol{\Xi} \qquad 5$$

The matrix $\boldsymbol{\Theta}(\boldsymbol{q}, \dot{\boldsymbol{q}}) \in \mathbb{R}^{3\times 14}$, is the function library containing candidate mechanisms describing COM accelerations, with seven candidate mechanisms per leg. Template Signatures were defined by the parameter matrix $\boldsymbol{\Xi}$.

Akaike Information Criterion (AIC)

In the following description of the AIC, we consider a nonlinear system with dynamics described by equation 5, where the estimated system dynamics are described by $\hat{f}(q)$, as the formulation below is not specific to models identified by Hybrid-SINDy.

The AIC is widely used to compare candidate models according to their number of free parameters (*i.e.*, model complexity), k, and log-likelihoods (*i.e.*, representativeness of the observations), $L(q, \hat{\mu})$. The AIC has the form:

$$AIC = 2k - 2\ln\big(L(q, \hat{\mu})\big). \qquad 6$$

Mangan and colleagues (2019) assumed that model errors are independently, identically, and normally distributed, enabling the AIC to be written in terms of the number of samples, ρ, and the sum of squared residuals [3]:

$$AAIC = 2k + \rho \ln\left(\frac{\sum_{j=1}^{\rho}\sum_{i=1}^{I}\big(\hat{f}(\mathrm{q}) - \dot{q}\big)_{i,j}{}^{2}}{\rho}\right), \qquad 7$$

where in the outer summation, P denotes the number of samples and in the inner summation, I denotes the number of output states. A correction may be applied to the AIC formulation to account for bias due to finite sample sizes (AICc):

$$AICc = AIC + \frac{2(k+1)(k+2)}{\rho - k - 2}. \qquad 8$$

The AICc score provides a level of support for each candidate model, with lower AICc scores indicating stronger support for a model. To compare competing models, a relative AICc score ($\Delta AICc$) may be calculated as the difference between the AICc score of the minimum-AICc model and that of competing models:

$$\Delta AICc_j = AICc_j - AICc_{min}, \qquad 9$$

where the subscript j, denotes the jth model. Burnham and Anderson [7] provided recommendations that $\Delta AICc \leq 2$ indicates strong support, while $\Delta AICc \leq 7$ indicates moderate support, $\Delta AICc$ values greater than seven have low support compared to competing models. Like Mangan and colleagues, we considered models with $\Delta AICc \leq 3$ to represent supported template signatures of walking [3].

Comparison to alternative modeling frameworks

Here we present a brief analysis comparing Hybrid-SINDy to alternative modeling dimensionality reduction frameworks: Stepwise regression[8] and principal components analysis (PCA).

Stepwise regression

Stepwise regression is similar to Hybrid-SINDy in that it identifies sparse system models, often by eliminating or adding terms until terms no longer improve accuracy or are statistically significant. However, unlike Hybrid-SINDy, stepwise regression only selects a single model, even if multiple models are equally plausible representations of the system[9]. Further, stepwise regression is known to select different models depending on the data, especially when predictor variables are colinear[9,10]. However, it is unclear if stepwise regression could produce similar models as Hybrid-SINDy.

Approach: To test this, we replaced the SINDy algorithm with a similar approach using stepwise regression. Our stepwise regression approach is similar to that of Shamaei and Colleagues (2013), who estimated ankle quasi-stiffness from mechanics-based functional forms[8].

First, to provide an example of how stepwise regression produces different and less-sparse results than SINDy, we compared stepwise regression, as described in[8], to SINDy for the synthetic SLIP without noise and with moderate noise (η = 1 mm). We report results for a single cluster in single-limb support.

Next, we applied a similar framework to human walking data and produced template signatures for walking in all three exoskeleton conditions, following the methodology described in the main manuscript.

Results: For synthetic SLIP data, Even in the noise-free condition, stepwise regression selected artificially high-dimensional dynamics (Table S1.1). The stepwise regression model had rank = 13, compared to the SINDy-based model, which identified 4 models ranging from 5- to 0-dimensional. Note that the SLIP model has at most 6 non-zero terms in each gait phase (*see Supplemental – S2, Table S2.1 for synthetic SLIP parameters*). With noise (Table S1.2), SINDy identified higher-dimensional solutions but also identified additional low-dimensional solutions that would likely be more plausible according to the AIC.

Table S1.1: Noise-free synthetic SLIP template signatures in one cluster in single-limb support.

Coefficient	Stepwise solution (rank = 13)	SINDy – model 1 (rank = 5)	SINDy – model 2 (rank = 4)	SINDy – model 3 (rank = 2)	SINDy – model 4 (rank = 0)
$k_{L,R}$	-244.8	-260.8	-221.7	-385.6	0
$k_{L,L}$	-359.4	-359.6	-345.1	0	0
$k_{L,R}L_{0,R}$	244.0	257.6	224.8	361.3	0
$k_{L,R}L_{0,L}$	252.5	251.9	223.1	0	0
$c_{L,R}$	-0.9	0	0	0	0
$c_{L,L}$	-14.9	-15.1	0	0	0
$k_{s,R}$	0.8	0	0	0	0
$k_{s,L}$	-0.6	0	0	0	0
$c_{s,R}$	0.0	0	0	0	0
$c_{s,R}$	0	0	0	0	0
$k_{f,R}$	0.8	0	0	0	0
$k_{f,L}$	-0.6	0	0	0	0

$c_{f,R}$	-0.0	0	0	0	0
$c_{f,R}$	0.0	0	0	0	0

Table S1.2: Synthetic SLIP template signatures with noise ($\eta = 1mm$) in one cluster in single-limb support.

Coefficient	Stepwise solution (rank = 11)	SINDy – model 1 (rank = 12)	SINDy – model 2 (rank = 11)	SINDy – model 3 (rank = 8)	SINDy – model 4 (rank = 3)
$k_{L,R}$	-455.0	-407.8	-219.4	32.6	41.0
$k_{L,L}$	0	-31.9	-112.7	-299.9	-269.0
$k_{L,R}L_{0,R}$	797.6	729.4	459.2	18.6	0
$k_{L,R}L_{0,L}$	-373.6	-325.3	-171.8	179.2	170.8
$c_{L,R}$	0	0	0	0	0
$c_{L,L}$	-8.8	-8.0	0	0	0
$k_{s,R}$	-306.8	-283.5	-192.7	0	0
$k_{s,L}$	217.5	199.4	128.4	0	0
$c_{s,R}$	-4.2	-4.2	-4.1	-3.8	0
$c_{s,R}$	-9.2	-9.3	-9.3	-8.8	0
$k_{f,R}$	-338.9	-315.6	-224.3	-51.9	0
$k_{f,L}$	260.6	242.2	169.8	0	0
$c_{f,R}$	-17.3	-17.4	-17.9	-15.3	0
$c_{f,R}$	0	0	0	0	0

In human data, stepwise regression also identified rank = 13 dynamics, while SINDy identified lower-rank dynamics (Table S1.3). Consistent with single-cluster results in the synthetic SLIP, stepwise regression-based template signatures were not sparse (Figure S1.2; compare to Figure 3 in the main manuscript). Figure S1.3 shows that the post-stroke template signatures are also not sparse, making it unclear which mechanisms are most important for template representations of CoM dynamics (compare to Figure 6 in the main manuscript).

Table S1.3: Example human template signatures in one cluster in single-limb support.

Coefficient	Stepwise solution (rank = 13)	SINDy – model 1 (rank = 12)	SINDy – model 2 (rank = 6)	SINDy – model 3 (rank = 2)	SINDy – model 4 (rank = 0)
$k_{L,R}$	-79.1	-65.3	-99.2	-150.6	0
$k_{L,L}$	18.9	5.1	11.8	0	0
$k_{L,R}L_{0,R}$	43.5	15.7	75.8	132.0	0
$k_{L,R}L_{0,L}$	10.1	35.1	0	0	0
$c_{L,R}$	0	0	0	0	0
$c_{L,L}$	-1.4	0	0	0	0
$k_{s,R}$	22.4	34.1	0	0	0
$k_{s,L}$	-19.3	-31.9	-13.8	0	0
$c_{s,R}$	-1.3	-0.7	1.1	0	0
$c_{s,R}$	-0.5	-0.3	0	0	0

$k_{f,R}$	29.6	44.4	16.5	0	0
$k_{f,L}$	-20.2	-31.3	-9.2	0	0
$c_{f,R}$	-1.0	0	0	0	0
$c_{f,R}$	0	0	0	0	0

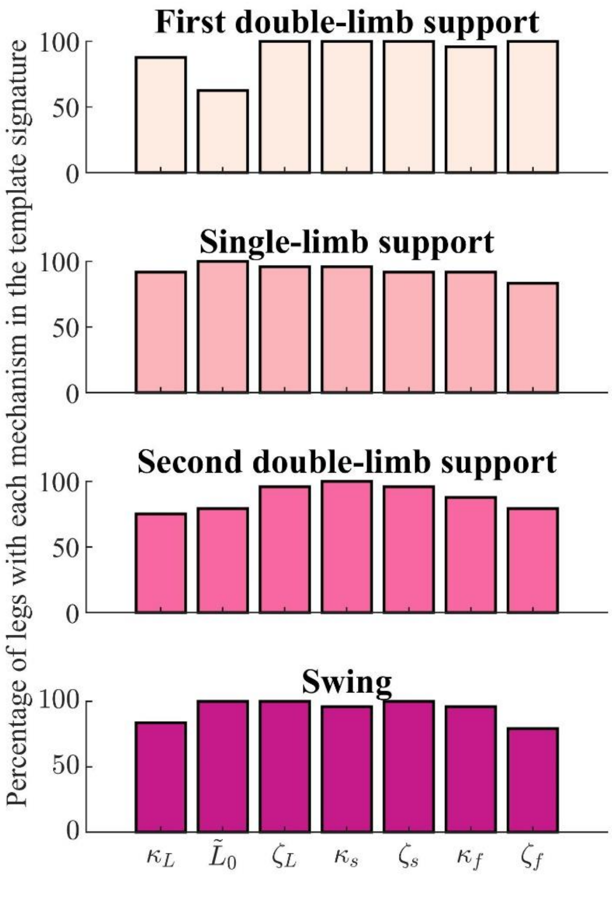

Figure S1.2: The percentage of unimpaired legs whose stepwise regression-based template signatures contained each mechanism in each gait phase. For 24 legs, the percentage of legs for which each template signature mechanism was selected by the Hybrid-SINDy algorithm. Colors denote each gait phase. Mechanisms selected in a larger percentage of legs suggest common representations of CoM dynamics, while less frequently selected mechanisms reflect individual-specific template features describing CoM dynamics.

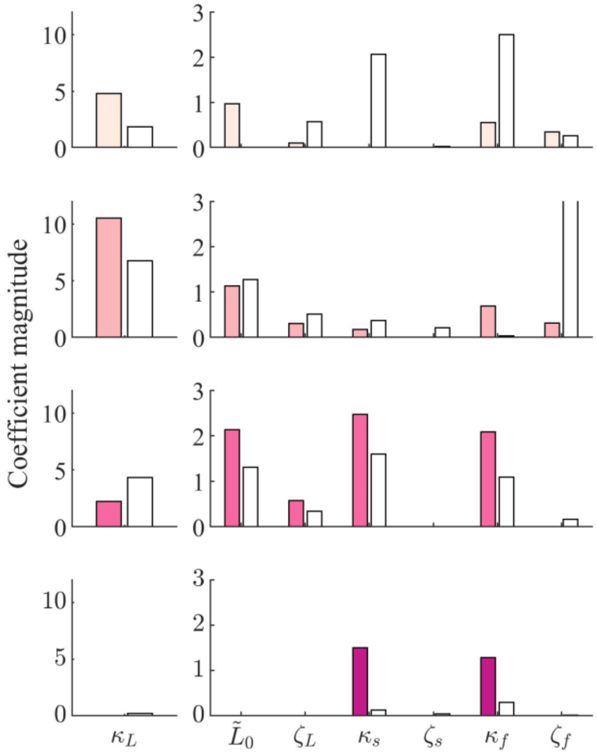

Figure S1.3: Inter-leg differences in template signatures in one stroke survivor identified using stepwise regression. Non-paretic (colored bars) and paretic (white bars) template signatures for one individual with post-stroke hemiparesis. Each plot represents a different gait phase.

Interpretation:
These results showcase that stepwise regression tends to select less parsimonious models than Hybrid-SINDy, hindering interpretation of template signatures. While regression hyperparameters may be tuned to reduce this dimensionality, Hybrid-SINDy's use of clustering and model selection within clusters likely enables more sparse template representations of CoM dynamics to be identified. Therefore, Hybrid-SINDy was appropriate for our work.

Principal components analysis (PCA)

Principal component analysis (PCA) could identify interpretable basis vectors describing CoM dynamics. For example, we could apply (e.g.) PCA or NMF to the nonlinear template states and use information criteria to select an optimal number of modes in the model[11].

However, PCA would not satisfy our goal of interpreting individual differences in template-based representations of CoM dynamics. PCA-based models would not be sparse in the space of template parameters: If the optimal (*e.g.*, according to the AIC) PCA or NMF-based model was unidimensional, the first PC would contain multiple template states. The higher-dimensional modes of these models would likely also contain multiple non-zero template states. Therefore, PCA would not provide the same mechanically intuitive interpretability as the sparse regression of template states used by Hybrid-SINDy.

Approach: To exemplify the limitation of PCA (with potential generalization to other dimensionality reduction approaches), we show that a sparse PCA-based model is still dense in the template mechanism space. Using data from our synthetic SLIP, we applied PCA to the template states, then used SINDy to identify the linear map, $\boldsymbol{\Xi}_{\mathbf{PC}}$, from PCs to CoM accelerations. We then projected the map back into the original template basis ($\boldsymbol{\Xi}_{\boldsymbol{template}}$ in Table S1.4**).**

Results: Table S1.4 shows an example of a 1-D linear map identified by SINDy. While the linear map is sparse in the PC space, it becomes dense when projected back into the original template mechanism space (Table S1.4).

Table S1.4: 1-dimensional PCA-based model of CoM dynamics.

PCA model term	1-D PC model ($\boldsymbol{\Xi}_{\mathbf{PC}}$)	1-D PC model projected into template state space ($\boldsymbol{\Xi}_{\boldsymbol{template}}$)
1	0	-0.417
2	0	-0.323
3	-0.351	-0.418
4	0	-0.323
5	0	-0.393
6	0	0.323
7	0	-0.136
8	0	0.172
9	0	-0.133
10	0	0.125
11	0	-0.185
12	0	0.106
13	0	-0.142
14	0	-0.183

Interpretation: Because—even for low-dimensional PC-based models—the resulting template model is full-dimensional, we cannot readily interpret which well-understood mechanisms are most important for describing CoM dynamics using template models. This limitation would likely generalize to other techniques like non-negative matrix factorization[12].

References

1 Holmes, P., Full, R. J., Koditschek, D. & Guckenheimer, J. The dynamics of legged locomotion: Models, analyses, and challenges. *SIAM review* **48**, 207-304 (2006).
2 Antoniak, G. *et al.* Spring-loaded inverted pendulum goes through two contraction-extension cycles during the single-support phase of walking. *Biology open* **8** (2019).
3 Mangan, N. M., Askham, T., Brunton, S. L., Kutz, J. N. & Proctor, J. L. Model selection for hybrid dynamical systems via sparse regression. *Proceedings of the Royal Society A* **475**, 20180534 (2019).
4 Kim, S. & Park, S. Leg stiffness increases with speed to modulate gait frequency and propulsion energy. *Journal of Biomechanics* **44**, 1253-1258, doi:https://doi.org/10.1016/j.jbiomech.2011.02.072 (2011).
5 Dingwell, J. B. & Cusumano, J. P. Identifying stride-to-stride control strategies in human treadmill walking. *PloS one* **10**, e0124879 (2015).
6 Dingwell, J. B. & Cusumano, J. P. Humans use multi-objective control to regulate lateral foot placement when walking. *PLoS computational biology* **15**, e1006850 (2019).
7 Anderson, D. & Burnham, K. Model selection and multi-model inference. *Second. NY: Springer-Verlag* **63** (2004).
8 Shamaei, K., Sawicki, G. S. & Dollar, A. M. Estimation of quasi-stiffness and propulsive work of the human ankle in the stance phase of walking. *PloS one* **8**, e59935 (2013).
9 Whittingham, M. J., Stephens, P. A., Bradbury, R. B. & Freckleton, R. P. Why do we still use stepwise modelling in ecology and behaviour? *Journal of animal ecology* **75**, 1182-1189 (2006).
10 Anderson, D. R. & Burnham, K. P. Avoiding pitfalls when using information-theoretic methods. *The Journal of wildlife management*, 912-918 (2002).
11 Schmid, P. J. Dynamic mode decomposition of numerical and experimental data. *Journal of fluid mechanics* **656**, 5-28 (2010).
12 Lee, D. D. & Seung, H. S. Learning the parts of objects by non-negative matrix factorization. *Nature* **401**, 788 (1999).

Supplemental – S2: Quantifying changes in individual-specific template-based representations of center-of-mass dynamics during walking with ankle exoskeletons using Hybrid-SINDy

Michael C. Rosenberg[1*], Joshua L. Proctor[1,2], Katherine M. Steele[1]

[1]Department of Mechanical Engineering, University of Washington, Seattle, United States of America
[2]Department of Applied Mathematics, University of Washington, Seattle, United States of America

Overview

This supplemental provides details of the simulations using a synthetic spring-loaded inverted pendulum (SLIP) dynamics to validate the Hybrid Sparse Identification of Nonlinear Dynamics (Hybrid-SINDy) for human walking.

5. Validating Hybrid-SINDy using a synthetic SLIP walking model
    a. Effects of measurement noise on algorithm performance
    b. Effects of missing physics on algorithm performance
6. Evaluation of collinearity of template signature state variables

Validating Hybrid-SINDy using a synthetic SLIP walking model

We evaluated Hybrid-SINDy's ability to accurately identify and select walking dynamics, using synthetic data generated from simulations of a bipedal SLIP walker with known dynamics (Table S2.5 and Figure S2.4)[1]. The bipedal SLIP had dynamics matching those used in human data (*Supplemental – S1*), with the addition of foot mass to enable simulation of a full gait cycle. The synthetic SLIP had asymmetric leg stiffness and damping, and masses representing the pelvis (M = 56 kg) and feet (M_f = 7 kg) (Table S2.5; Figure S2.4). We omitted rotary mechanisms from the SLIP dynamics but used the full function library described in *Supplemental – S1*, equation 4. To replicate the human walking datasets, we simulated 120 gait cycles each from randomly perturbed initial conditions with an average initial velocity of 1.20 m/s. The SLIP walked at approximately 1 stride/s.

Table S2.5 – Bipedal SLIP normalized simulation parameters.

Term	***[Right leg, Left leg]***			
	**DS$_1$**	**SS**	**DS$_2$**	**SW**
κ_L	[10.8, 9.1]	[25.1, 18.82]	[9.03, 10.68]	[36.6,25.3]
$\tilde{L}_0$	[1.0, 1.0]	[1.0, 0.7]	[1.0, 1.0]	[0.7, 0.7]
ζ_L	[0.04, -0.07]	[-0.03, -0.04]	[0, 0]	[-0.40, -0.57]
κ_s	0	0	0	0
ζ_s	0	0	0	0
κ_f	0	0	0	0
ζ_f	0	0	0	0

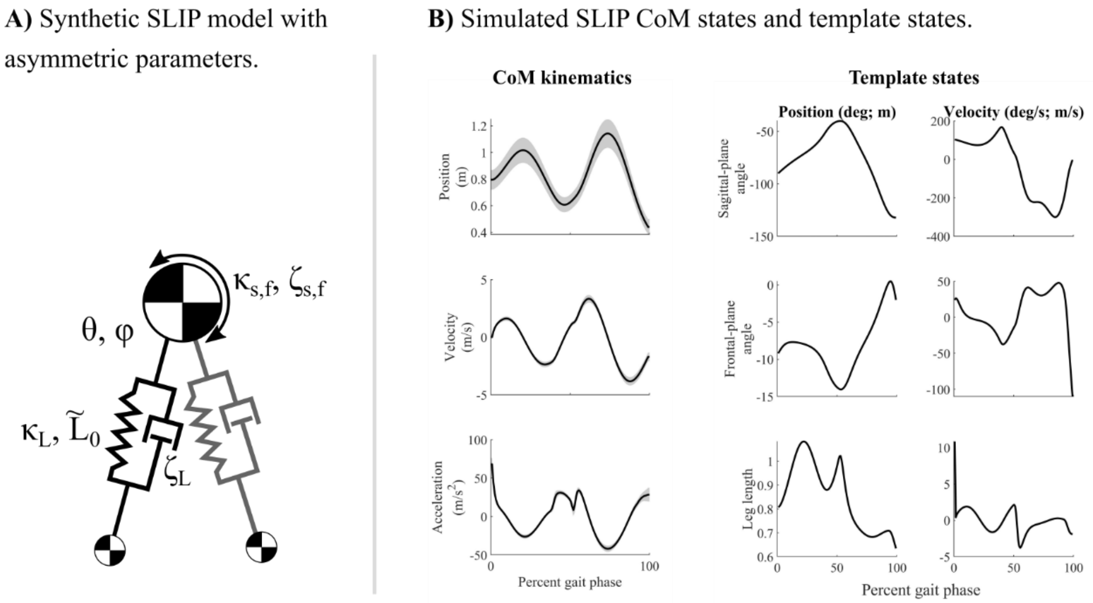

Figure S2.4: Synthetic SLIP model depiction and simulated CoM states. A) Two-dimensional depictions of the simulated SLIP. The simulated SLIP used leg springs and dampers, as well as foot masses to simulate full strides. B) Time-series measurements of simulated CoM position, velocity, and acceleration for the simulated SLIP (left; average ± 1SD). Template states (right) show trajectories of the template variables used in the function library to estimate template signature coefficients (average ± 1SD).

We conducted two analyses using the simulations of a synthetic SLIP model: First, we evaluated the effects of measurement noise on Hybrid-SINDy's ability to estimate synthetic SLIP parameters in the presence of sensor noise. Second, we evaluated Hybrid-SINDy's ability to estimate synthetic SLIP parameters when a subset of the terms constituting the true system dynamics is missing from the function library.

Effects of measurement noise on algorithm performance

Approach: As SINDy's performance is sensitive to sensor noise, we identified template signatures using simulation measurements with Gaussian noise ranging logarithmically from 10 *nm* to 10 *cm* added to the position measurements[2]. After adding noise, we differentiated each gait cycle separately before concatenating the strides to avoid errors in the estimated time derivatives due to changes in the initial conditions of each simulation. We quantified the Hybrid-SINDy's ability to reconstruct model dynamics using the number of correctly selected mechanisms and the accuracy of estimated coefficients. We also evaluated the ability of each model to predict the ground truth (noise-free) CoM accelerations.

Results: In the noise-free condition, the Hybrid-SINDy algorithm correctly identified the salient features of the simulated SLIP's template signature during single-limb support (Figure S2.5). Specifically, Hybrid-SINDy identified non-zero leg stiffness (κ_L), resting length ($\tilde{L}$), and damping (ζ_L) terms only – consistent with the SLIP's dynamics. In the noise-free condition, 86% of terms were correctly selected and only one term, leg damping in first double-limb support, was incorrectly selected (Figure S2.5B). Both double-limb support phases were very short in the simulated SLIP: 1.3% and 4.6% of samples in first and second double-limb support – smaller than the 7.4% of the training data in each cluster. Consequently, all clusters with centroids in the double-limb support phases contained samples spanning double- and single-limb support. Hybrid-SINDy incorrectly omitted only damping terms with small coefficients (Figure S2.5). In single-limb support and swing, coefficients were within 2% of the ground truth values. While prediction accuracy (averaged across output dimensions) was lower than expected ($r^2 = 0.88$), errors occurred primarily at the transitions between gait phases and in the double-limb support phases (Figure S2.5A, left: deviations in the predictions (colors) relative to ground truth simulation results (gray); transitions occur where colors change).

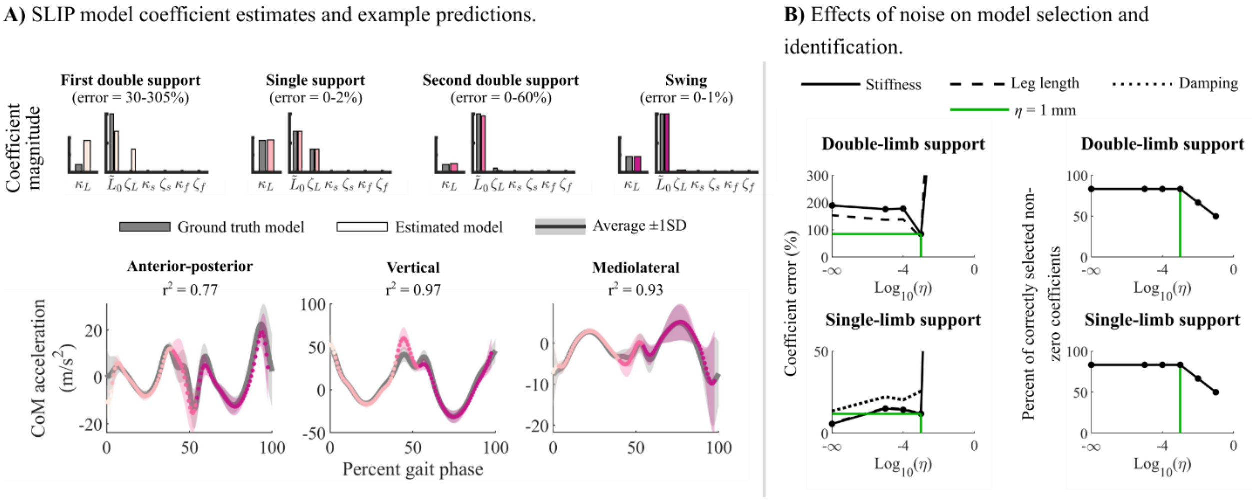

Figure S2.5: Parameter estimates, prediction accuracy of simulated CoM kinematics, and the effects of noise on model identification accuracy. A) Model coefficients (top) can be compared between walking conditions. Bar plots show ground truth synthetic SLIP parameters (gray) and the corresponding estimated template signature coefficients (colors). The range of percent errors in coefficients is shown above each plot. The average (±1SD) ground truth CoM accelerations of the synthetic SLIP (bottom; gray) and model predictions in each gait phase (colors). Model accuracies are quantified using the

coefficient of determination (r^2). B) Effects of noise on model coefficient percent error (left) and the percent of correctly selected mechanisms (right). All horizontal axes represent noise magnitudes on a log scale. For coefficient error, percent errors were computed for stiffness (solid line), leg length (dashed line), and damping (dotted line) separately. Green lines denote the η = 1 mm noise level.

When measurement noise with a standard deviation of 1 mm—similar to that of our motion capture system—was added to the system measurements, Hybrid-SINDy correctly selected 83% of mechanisms, with incorrect mechanisms selection occurring primarily in double-limb support. Template signature coefficients were estimated with an average error of less than 29% in single-limb support and swing (**Figure S2.5B**). In single-limb support, Hybrid-SINDy was still able to estimate large coefficients (*i.e.*, leg stiffness and resting length) with less than 1% error. Even at this noise level, the estimated template signature could still predict the noise-free data with similar accuracy to the noise-free data ($r^2 = 0.83$). The accuracy of coefficient estimates and model predictions rapidly declined at noise levels with standard deviations larger than 1 mm.

Interpretation: Hybrid-SINDy's ability to identify template signatures from forward simulations of a synthetic SLIP model with known dynamics at measurement noise levels comparable to marker-based motion capture suggests that individual differences in template signatures of human gait data likely reflect some differences in the underlying CoM dynamics. Hybrid-SINDy performed as expected in the noise-free condition, accurately identifying single-limb support and swing dynamics, except for small damping terms. Terms with small contributions to system behavior, such as damping in this study, may be omitted by the AICc in favor of parsimony[3,4]. Therefore, Hybrid-SINDy will not always select the true system dynamics but will select terms critical to describing the salient aspects of CoM dynamics. In our human walking data, Hybrid-SINDy may, therefore, omit mechanisms describing subtle idiosyncrasies in CoM dynamics that could be important for gait stability or efficiency. Future experiments using different constraints or perturbations may identify additional mechanisms needed to describe CoM dynamics.

The reduced accuracy of synthetic SLIP template signatures in double-limb support and with increasing noise magnitude is consistent with the results of Hybrid-SINDy applied to a spring-mass hopper[5]. However, accurate estimates of single-limb support CoM dynamics and longer double-limb support times in human walking data support our use of 800 samples per cluster for human template signatures. Longer time-series datasets would help to ensure that clusters are small enough to span only a single gait phase but large enough to remain robust to sensor noise.

Effects of missing physics on algorithm performance

Approach: We tested the robustness of estimated template signature coefficients using simulations of a synthetic SLIP: We simulated SLIP walking at speeds ranging from 1.00-1.75 m/s (Froude speeds: 0.10-0.31) using identical dynamics across all simulations. Next, we confirmed that, in the noise-free condition, Hybrid-SINDy identified the same model coefficients for all simulations when using the *complete* function library that contained all functional forms constituting the SLIP's dynamics. To characterize how missing functional forms affected Hybrid-SINDy's ability to identify model coefficients, we applied Hybrid-SINDy to the same data but with the left- and right-leg axial damping terms removed from the function library. We quantified the effect of missing physics (damping terms) as the percent differences in the estimated model coefficients (averaged across coefficients) between simulations. We defined the similarity of kinematics between simulations as the root-mean-squared (RMS) difference between phase-averaged kinematics.

Results: The kinematic differences observed in SLIP simulations were larger than those seen in our human data (Figure S2.6A, shaded boxes represent the range of human data). We observed this difference despite participants walking at self-selected speeds ranging from 1.20-1.50 m/s (Froude speeds: 0.16-0.29), comparable to the range tested *in silico*. For *within-participant* comparisons, differences in kinematics were smaller than those observed in any simulation pairs (Figure S2.6A, blue shaded region; Figure S2.6C).

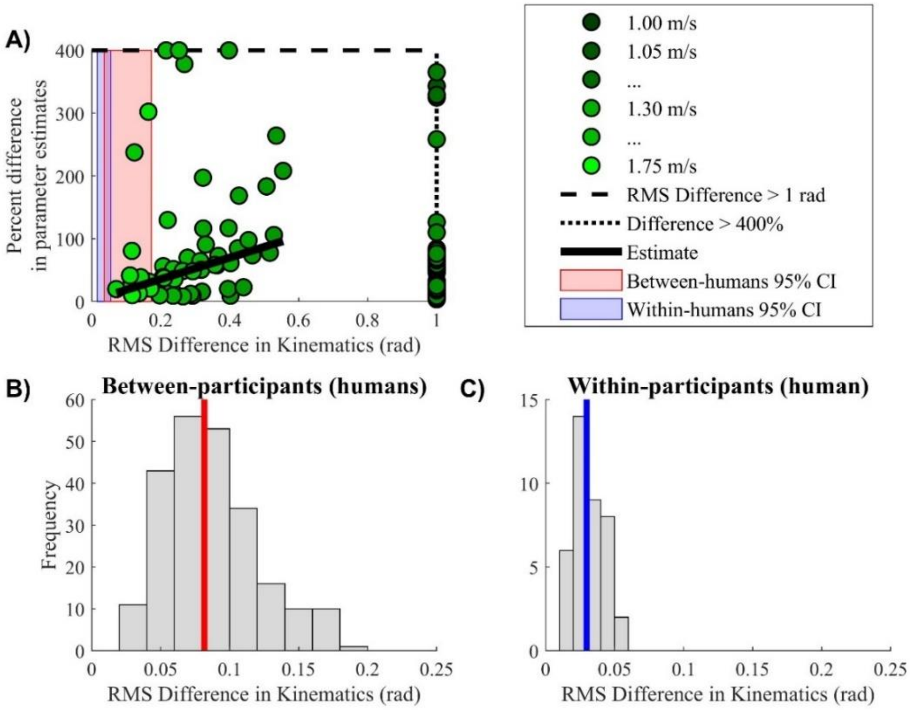

Figure S2.6: Regression analysis of differences in estimated synthetic SLIP coefficients as a function of differences in kinematics, and compared to differences in human kinematics. Pair-wise differences in estimated coefficients and kinematics between simulations was computed as the root-mean-squared difference across coefficients or the stride-averaged kinematic trajectories, respectively. Note that the model coefficients were normalized, such that the differences in coefficients are dimensionless. Also note that we fixed template signature structure to match that of the signature selected for the 1.35 m/s

simulation. A) Regression analysis of synthetic data using the 10 fastest speeds (1.30-1.75 m/s). Green dots denote simulation pairs. Some simulation pairs had large differences due to near-zero coefficient estimates; their values are truncated on the vertical axis at 400% (dashed line). Simulations at speeds under 1.30 m/s had large differences in kinematics; their values are truncated on the horizontal axis (dotted line). The shaded regions represent 95% confidence intervals of differences in kinematics in the human data between- (red) and within-participants (blue). These colors correspond to the colors of vertical lines in panels B and C. B) Histogram of between-participants differences in kinematics in the human data. Each sample represents a pair of participants in the same walking condition. The red line represents the median difference in kinematics. C) Histogram of within-participants differences in kinematics in human data. Each sample represents a pair of exoskeleton conditions (e.g., shoes-only vs. zero-stiffness) for the same participant. The blue line represents the median difference in kinematics.

When the *complete* function library was included, Hybrid-SINDy identified nearly consistent template signatures across all walking speeds. In single-limb support and swing, the estimated coefficients were consistent across simulations; differences in leg stiffness and resting length estimates were 3.9 ± 4.5% and 0.7 ± 0.9%. Similar to our analysis of noise, estimated coefficients were more variable in double-limb support (differences > 269%), likely because the clusters were larger than the simulated double-limb support phases.

Compared to using the complete function library to identify template signatures, *missing physics* resulted in larger differences in estimated coefficients across the full speed range. When the same term was active in two simulations, the differences in leg stiffness and resting length estimates were 6.3 ± 5.2% and 1.3 ± 1.4%, respectively, in single-limb support. Unsurprisingly, differences were larger in double-limb support (>>25%) as double-limb support phases were short compared to the cluster sizes. Different template signature structures (i.e., different non-zero terms) were selected across simulations, making this analysis similar to a *between-participants* comparison.

Because our analysis of human data used a single (shoes-only) template signature structure for *within-participants* comparisons, we conducted a second analysis to reflect this comparison: We fixed the synthetic SLIP template signature structures to the structure selected for an intermediate speed (1.35 m/s) and estimated model coefficients. Compared to template signatures with unique structures identified for each simulation, the differences in axial leg stiffness and resting length estimates were only marginally reduced: 5.7 ± 5.0% and 0.5 ± 0.5%, respectively, in single-limb support and remained larger in double-limb support.

In this within-participants analysis, missing physics resulted in differences in coefficients driven by changes in simulated kinematics. At speeds slower than 1.30 m/s, differences in the estimated coefficients were not correlated with differences in kinematics. However, within the 1.30-1.75 m/s speed range, larger differences in simulated kinematics were associated with larger differences in coefficient estimates ($r^2 = 0.50$, $p < 0.001$; Figure S2.6A, black estimate line).

Interpretation: Small differences in kinematics in humans compared to the synthetic SLIP simulations suggest that large differences in template signature coefficients are unlikely due entirely to differences in kinematics. However, at least 5-10% differences in template signature coefficients can be attributed to changes in kinematics rather than CoM dynamics. Larger differences in coefficient estimates may be needed for human data, depending on how well the model captures the underlying dynamics.

Covariation of template signature state variables

Covariation among the state variables corresponding to mechanisms in the Hybrid-SINDy function library can hinder the interpretation of template signatures. Specifically, covariation in state variables can alter coefficient estimates if both mechanisms corresponding to two co-varying states are selected, or if redundant mechanisms are selected at random for different participants.

Approach: To investigate the effect of covariation between state variables for the human data, we correlated SLIP states within each gait phase separately (Figure S2.7; average coefficient of determination across participants).

Results: Leg stiffness and resting length were most strongly correlated, as expected ($r^2 = 0.99$). These states were both selected by Hybrid-SINDy. Most correlations were less than $r^2 = 0.70$ (Figure S2.8 highlights strong correlations: $r^2 > 0.70$). While frontal-plane rotary stiffness (κ_f) and damping (ζ_f) in double-limb support were strongly correlated, only frontal-plane rotary stiffness was selected for a subset of participants (see Figure 3, main manuscript).

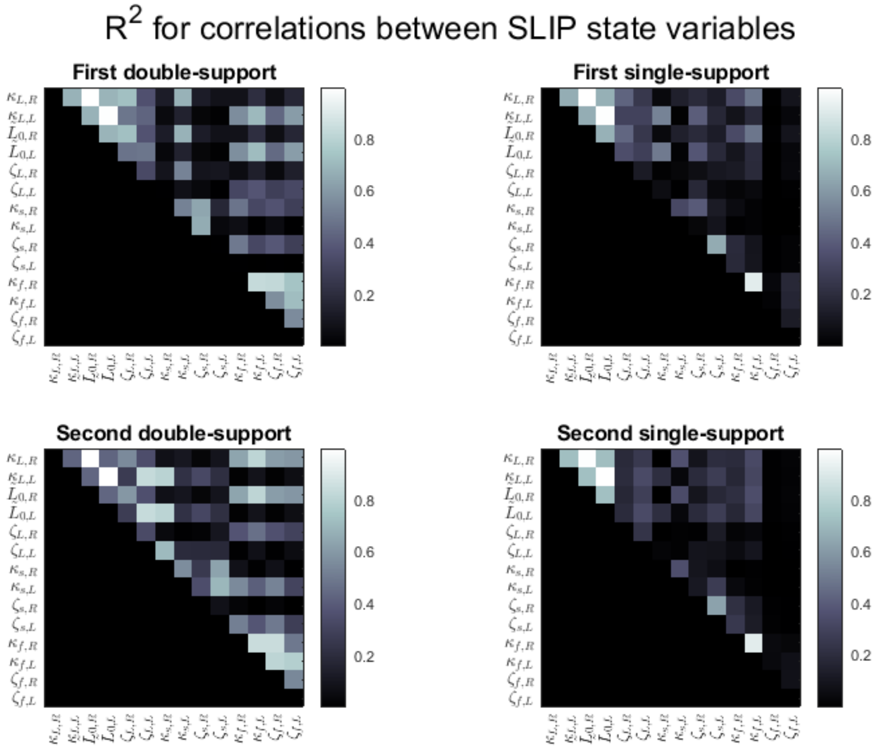

Figure S2.7: Correlations between SLIP states in human walking data for each gait phase. Colormaps of R-squared (R^2) values denoting correlations between each pair of state variables in each gait phase (one panel for each phase, denoted by titles above each panel). Black boxes denote $R^2 = 0$ and white boxes denote $R^2 = 1$. The lower triangle is omitted for clarity (the correlation matrix is symmetric). Along the axes, the second subscript on each variable denotes the right (R) or left (L) leg. Only axial stiffness (κ_L) and leg resting length (L_0) states were, as expected, very strongly correlated.

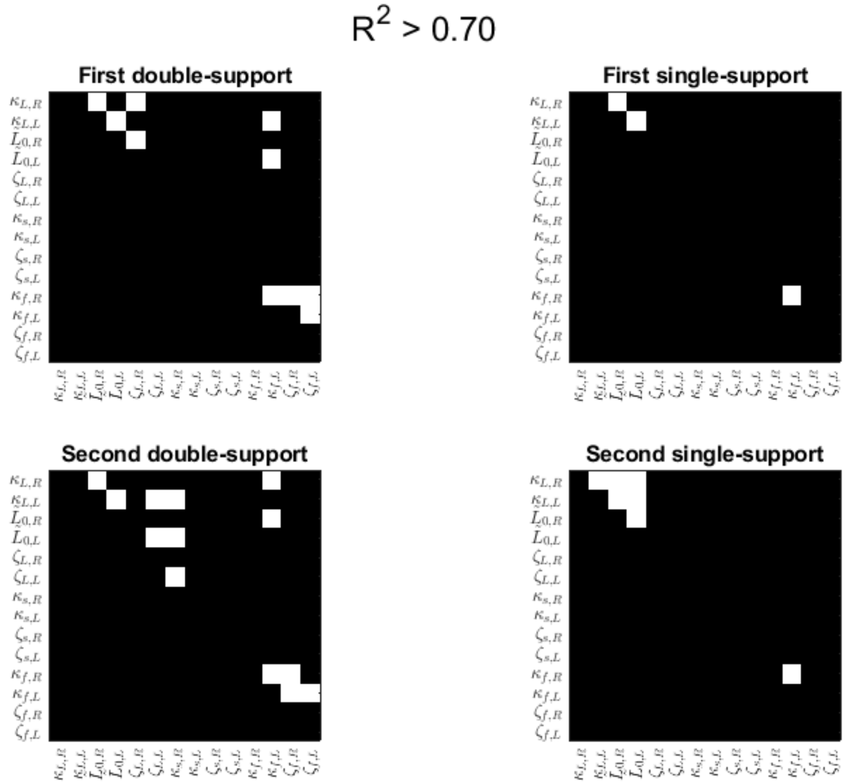

Figure S2.8: Strong correlations between SLIP states in human walking data for each gait phase. Colormaps of R-squared (R^2) values highlight strong correlations ($R^2 > 0.70$) between each pair of state variables in each gait phase (one panel for each phase, denoted by titles above each panel). Black boxes denote $R^2 \leq 0$ and white boxes denote $R^2 > 0.70$. The lower triangle is omitted for clarity (the correlation matrix is symmetric). Along the axes, the second subscript on each variable denotes the right (R) or left (L) leg. While frontal-plane rotary stiffness (κ_f) and damping (ζ_f) in double-limb support were strongly correlated, only frontal-plane rotary stiffness was selected for some participants. However, Hybrid-SINDy consistently selected frontal-plane rotary stiffness, suggesting that stronger correlations are needed to select different mechanisms for different participants.

Interpretation: Hybrid-SINDy's performance appears robust to moderate correlations in the state variables. Covariation in the state variables did not result in unexpected variables being included in the template signatures. However, different combinations of parameters could have similar statistical plausibility. In this case, our use of the Akaike Information Criterion and multi-model inference acknowledges the existence of multiple plausible template signatures[3,4]. While we selected mechanisms with distinct relationships to CoM accelerations, studies using larger or non-mechanistic function libraries may be more strongly impacted by covariation in the state variables. Such studies should evaluate covariation among state variables before model fitting.

References

1 Geyer, H., Seyfarth, A. & Blickhan, R. Compliant leg behaviour explains basic dynamics of walking and running. *Proceedings of the Royal Society B: Biological Sciences* **273**, 2861-2867 (2006).

2 Brunton, S. L., Proctor, J. L. & Kutz, J. N. Discovering governing equations from data by sparse identification of nonlinear dynamical systems. *Proceedings of the National Academy of Sciences* **113**, 3932-3937 (2016).

3 Akaike, H. in *Selected Papers of Hirotugu Akaike* 215-222 (Springer, 1974).

4 Anderson, D. & Burnham, K. Model selection and multi-model inference. *Second. NY: Springer-Verlag* **63** (2004).

5 Mangan, N. M., Askham, T., Brunton, S. L., Kutz, J. N. & Proctor, J. L. Model selection for hybrid dynamical systems via sparse regression. *Proceedings of the Royal Society A* **475**, 20180534 (2019).
